# The protective effects of nesfatin‐1 in neurological dysfunction after spinal cord injury by inhibiting neuroinflammation

**DOI:** 10.1002/brb3.2778

**Published:** 2022-10-21

**Authors:** Xin Jin, Kai Guan, Zhengyu Chen, Yongwei Sun, Hongjun Huo, Jinle Wang, Huihui Dong

**Affiliations:** ^1^ Department of Orthopedics II the First People's Hospital of Xianyang Xianyang Shaanxi China

**Keywords:** inflammation, nesfatin‐1, neurological dysfunction, NF‐κB, spinal cord injury

## Abstract

**Aims:**

Spinal cord injury (SCI) is one of the most severe neurological diseases. However, there is still no effective treatment for it. Nesfatin, a precursor neuropeptide derived from nucleobindin 2 (NUCB2), has displayed a wide range of protective effects in different types of cells and tissue. However, the effects of nesfatin‐1 in SCI have not been reported before.

**Materials and methods:**

A SCI model was established. The behavior of mice was assessed using the Basso, Beattie, and Bresnahan (BBB) assessment.

**Results:**

Here, we report that the administration of nesfatin‐1 improved neurological recovery in SCI mice by increasing BBB scores, reducing lesion area volume and spinal cord water content. Also, nesfatin‐1 ameliorated oxidative stress by reducing reactive oxygen species (ROS) levels and increasing superoxide dismutase (SOD) activity. We also found that nesfatin‐1 prevented neuronal apoptosis in SCI mice by reducing caspase 3 activity and the expression of Bax, as well as increasing B‐cell lymphoma‐2 (Bcl‐2). Additionally, nesfatin‐1 reduced the levels of interleukin 6 (IL‐6), interleukin‐1β (IL‐1β), and tumor necrosis factor‐α (TNF‐α). Nesfatin‐1 also promoted microglia towards M2 polarization by increasing the marker CD206 but reducing CD16. Importantly, nesfatin‐1 enhanced the phosphorylation of signal transducer and activator of transcription 1 (STAT1) but reduced the expression levels of toll‐like receptor 4 (TLR4) and phosphorylated nuclear factor kappa‐B p65 (p‐NF‐κB p65).

**Conclusion:**

Our findings imply that nesfatin‐1 exerts neuroprotective actions in SCI by promoting the activation of M2 microglia, and its underlying mechanisms might be related to the activation of STAT1 and inhibition of the TLR4/NF‐κB signaling pathway.

## Graphical abstract

Nesfatin‐1 prevented neuronal apoptosis in mice underwent SCI;

Nesfatin‐1 prevented activation of NF‐?B signaling but increased phosphorylation of STAT1;

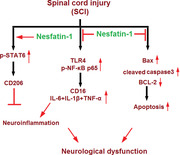

## 1 INTRODUCTION

Spinal cord injury (SCI) refers to damage to the spinal cord structure or function induced by trauma, tumors, infection, malformations, or iatrogenic factors, which seriously affect the life quality of patients. Bleeding, local shock, hypoxia, adenosine triphosphate reduction, microglial activation, and proinflammatory cytokines release are the main pathological characteristics of SCI. The morbidity of SCI in China ranges from 15 to 60 per million and shows an increasing trend annually (Ramos et al., 2016). According to the pathologic process, SCI can be divided into primary injury and secondary injury. The primary damage is instantaneous and irreversible, while the secondary injury develops on the basis of primary injury and its pathological process includes demyelination, axon and neuron necrosis, and other reversible cellular tissue damage (Anwar et al., 2016). Microglia are macrophages widely located in the nervous system and are involved in the cellular immune process of the nervous system. Under normal circumstances, spinal microglia are in a resting state, in which they monitor pathological responses timely (Papa et al., 2014) and play a role in immune monitoring. After SCI, microglia go through a series of morphological and functional changes from their resting state and subsequently polarize into M1 and M2 types to perform their respective functions. M2 microglia, represented by the overexpression of CD206, can be divided into three subtypes, M2a, M2b, and M2c, which are stimulated by different cytokines, respectively and contribute to the repairing of nerve cells and regeneration of nerve tissues (Lim et al., 2017). Overactivated M1 microglia, represented by the overexpression of CD16, secrete a large number of inflammatory factors, cytokines, and free radicals, such as colony‐stimulating factor (M‐CSF), TNF‐α, interleukin‐1 (IL‐1), and interleukin‐6 (IL‐6), which induce serious inflammatory reactions and inhibit myelin regeneration (Saxena et al., 2015). M1 microglia can be stimulated by lipopolysaccharides (LPS) or interferon γ (IFN‐γ), while M2 microglia can be stimulated by cytokines such as interleukin‐4 (IL‐4), interleukin‐10 (IL‐10), and transforming growth factor α (TGF‐α). Microglia are in a classic dynamic balance between M1 and M2 polarization, and their transformation is closely associated with the local microenvironment in the development of SCI (Lee et al., 2016). Therefore, regulating the M1/M2 polarization state balance to avoid the occurrence of neuroinflammatory reactions is of great value and significance for protecting the nervous system from secondary injury, and also plays an important role in the diagnosis and treatment of SCI.

Nucleobindin 2 (NUCB2) is encoded by the NEFA gene and consists of a 24‐amino acid polypeptide and 396‐amino acid protein. The sequence of the NUCB2 amino acid is highly homologous among humans, mice, and rat species. NUCB2 can be cleaved into three segments, nesfatin‐1, nesfatin‐2, and nesfatin‐3 by the prohormone invertase, among which nesfatin‐1 can be expressed in humans, pigs, fish, mice, canine, and other biological populations (Gaige et al., 2013; Gonkowski et al., 2012). It is mainly distributed in the paraventricular nucleus (PVN), supraoptic nucleus, arcuate nucleus, solitary tract nucleus, and lateral region (LHA) in the central system (Foo et al., 2008). Nesfatin‐1 is reported to cross the blood–brain barrier in an unsaturated manner and its central and peripheral distribution is affected by the intertransmission between serum and cerebrospinal fluid (Pan et al., 2007). Recently, promising anti‐inflammatory properties are reported for nesfatin‐1 (Meng et al., 2021; Wang et al., 2020). However, it is unknown whether nesfatin‐1 possesses a protective effect against SCI. Here, we aim to assess the possibility of treating SCI with nesfatin‐1 by examining the effects of nesfatin‐1 on SCI animals and exploring the underlying mechanism.

## 2 MATERIALS AND METHODS

### 2.1 The establishment of SCI in mice

The protocols of this study were approved by the Ethical Committee of the First People's Hospital of Xianyang (XYH‐EA20190A021). C57bl/6 mice were obtained from SLRC Laboratory Animal for the establishment of the SCI model. Forty‐eight mice were used in the experiments, 15–16 mice were used for each group, and one mouse died in surgery. Pentobarbital sodium was injected intraperitoneally into the mice, who were then secured to the surgical table. Neck skin was incised, and the fascia and muscles on both sides were bluntly separated with forceps until the cervical lamina was exposed. The C5 lamina was removed with pliers, followed by puncturing the pia mater spinalis of the posterior horn of the spinal cord using the No. 26th acupuncture. The dorsal corticospinal tract was injured twice with the modified No. 5th FSTDUMONT forceps, which were inserted into the dorsal horn gray matter along the part of the dorsal horn gray matter (1 mm). Then, the tip of one side of the modified No. 5th FSTDUMONT forceps was inserted into the dorsal pinhole of the right spinal cord (0.8 mm), and the tip of the other side was left outside the spinal cord. The red nucleus spinal tract and corticospinal lateral tract were clamped for 15 s, twice in total. After the surgery, neck muscles and skin were sewn together, and animals were disinfected and kept warm until they woke up. In the Sham group, cervical muscle and skin were directly sutured without operations after cervical lamina exposure.

### 2.2 In vivo animal studies and nesfatin‐1 administration

Animals were divided into three groups: the Sham, SCI, and SCI + nesfatin‐1. Animals in the SCI + nesfatin‐1 group were intraperitoneally injected with nesfatin‐1 at a dose of 20 μg/kg daily for 7 days. Mice in the Sham and SCI group were intraperitoneally injected with the same volume of normal saline. Each of these groups had four subgroups (1‐, 3‐, 7‐, 14‐, 21‐, and 28‐day time points).

### 2.3 Basso, Beattie, and Bresnahan assessment

The behavior of mice was evaluated at 1, 3, 7, 14, 21, and 28 days post SCI modeling, and a scale of 0–21 score was graded on the Basso, Beattie, and Bresnahan (BBB) test. Severe neurological deficits were represented by 0 point, and normal condition was indicated by 21 points. The detailed evaluation method was referring to the description reported previously (Z. He et al., 2017; H.‐T. Li et al., 2016). Operators of BBB are blinded to the group information.

### 2.4 Dihydroethidium staining

ROS levels in the SCI area were examined using dihydroethidium (DHE) staining. The frozen sections of the SCI area were cut into 5‐μm thick with a CM1950 frozen slicer, which were then incubated with DHE in the dark at 37°C for 30 min. After washing with PBS, ROS levels were assessed using an Eclipse 55I fluorescence microscope. Image‐pro Plus 6.0 was used to analyze the intensity of ethidium bromide fluorescence to evaluate the levels of ROS in SCI areas.

### 2.5 Immunofluorescence analysis of cleaved caspase‐3

The SCI area was rinsed to remove residual fixative and impurities, followed by dehydration, waxing, embedding, and slicing. Antigen was then repaired after section de‐waxing. The section was rinsed with PBS three times, and a rabbit anti‐mouse‐cleaved caspase‐3 antibody (Cat# ab214430, 1:200, Abcam, UK) was added to be incubated at 4°C overnight in the dark. After being rewarmed for 30 min on the next day, a goat anti‐rabbit IgG fluorescent secondary antibody (Cat#ab150078, 1:500, Abcam, UK) was added and cultured at 37°C for 1 h. Slides were observed under a microscope (Leica, Germany).

### 2.6 Spinal cord water content assay

Mice were anesthetized with an intraperitoneal injection of 30 mg/kg 2% pentobarbital sodium, followed by stripping paravertebral muscles and quickly removing the spinal cord tissue. With the spinal cord injury site as the center, 0.5 cm was taken from the cephalic and caudal sides. The wet spinal cord specimens were immediately weighed. After being dried for 48 h in an oven at 80°C, the dry spinal cord specimens were weighed. The mean value was taken after repeated measurements three times to calculate the spinal cord tissue water content.

### 2.7 SOD activity

The activity of SOD (E‐BC‐K022‐M, Elabscience, USA) and caspase‐3 (E‐EL‐M0238c, Elabscience, USA) in the SCI area was detected with commercial kits using the hydroxylamine method and ELISA method according to the introduction description, respectively.

### 2.8 Enzyme‐linked immunosorbent assay

The concentrations of TNF‐α, IL‐6, and IL‐1β in the SCI area were detected utilizing enzyme‐linked immunosorbent assay (ELISA) (R&D Systems, USA). After collecting the supernatant of tissue homogenate following centrifugation, the 96‐well plate was implanted with the supernatant and the standards, respectively. Conjugate reagents were introduced into the system following 90‐min incubation. Then, the 3,3′,5,5′‐Tetramethylbenzidine (TMB) solution was added for 15 min incubation, followed by adding the stop solution. Lastly, a microplate reader (BMG LABTECH, Germany) was used to obtain the absorbance at 450 nm.

### 2.9 Real‐time PCR assay

RNAs were isolated from the SCI area with the TRIzol reagent, followed by transcribing into cDNA with a 1st‐Strand HiScript II Q RT SuperMix for qPCR (+gDNA wiper) Kit (Vazyme, China). The ChamQ Universal SYBR qPCR Master Mix (Vazyme, China) was used for the conduction of the PCR reaction. And the 2^−ΔΔCt^ method was utilized for the calculation of gene levels. The primer sequences were listed as follows: CD16 (F: 5′‐CAGGAAACAGCTATGACCTCCC‐3′, R: 5′‐TCCTCAGGTGAATAGGGTCTTC‐3′); CD206 (F: 5′‐TTCAGCTATTGGACGCGAGG‐3′; R: 5′‐GAATCTGACACCCAGCGGAA‐3′); GAPDH (F: 5′‐AGCCACATCGCTCAGACAC‐3′, R: 5′‐GCCCAATACGACCAAATCC‐3′).

### 2.10 Western blot analysis

The SCI areas were lysed using the lysis buffer to obtain total proteins, which were quantified with the bicinchonininc acid (BCA) method (Elabscience, USA). The sodium dodecyl sulfate polyacrylamide gel electrophoresis (SDS‐PAGE) was used to separate proteins at a 12% concentration and proteins were further transferred to the polyvinylidene fluoride (PVDF) membrane (GE Healthcare Life, USA). After blocking, primary antibodies against Bax (Cat#GB11007, Servicebio, China, 1:1000), Bcl‐2 (Cat#AF6139, Affinity, USA, 1:1000), CD206 (Cat# ab300621, Abcam, UK, 1:2000), CD16 (Cat# ab203883, Abcam, UK, 1:2000), p‐STAT1 (Cat#AF3300, Affinity, USA, 1:1000), TLR4 (Cat#AF7017, Affinity, USA, 1:1000), NF‐κB p65 (#AF5006, Affinity, USA, 1:1000), p‐NF‐κB p65 (Cat#AF2006, Affinity, USA, 1:1000), and β‐actin (Cat#4970, CST USA, 1:10,000) were added to be incubated at 4°C for 12 h. Subsequently, the PVDF membrane was incubated with the secondary antibody (Cat# ab6721, Abcam UK, 1:5000) for 1.5 h, followed by quantifying the level of target proteins by analyzing bands with the Image J software.

### 2.11 Statistical analysis

Data were expressed as mean ± standard deviation (SD), which was analyzed utilizing the one‐way analysis of variance (ANOVA) method followed by the Tukey's test. Statistical significance was determined when the value of *P* was lower than .05.

## 3 RESULTS

### 3.1 Nesfatin‐1 improved neurological recovery in SCI mice

**FIGURE 1:**
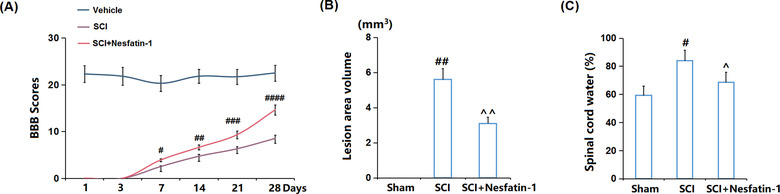
Nesfatin‐1 improved neurological recovery in SCI mice. (a) Basso, Beattie, and Bresnahan (BBB) scores at 1, 3, 7, 14, 21, and 28 days; (b) Lesion area volume; (c) Spinal cord water contents on 28 days after SCI (^#,##,###,####^ *P* < .05, .01, .005, .001 vs. vehicle group; ^,^^*P* < .05, .01 vs. SCI group)

Firstly, the neurological recovery of SCI mice was evaluated. The BBB scores (Figure 1a) at 1, 3, 7, 14, 21, and 28 days in SCI mice postmodeling were dramatically declined compared to the Sham group. After treatment with nesfatin‐1, the BBB scores were greatly elevated from 2.5 to 3.9, 4.7 to 6.6, 6.3 to 9.3, and 8.5 to 14.6 at 7, 14, 21, and 28 days postmodeling, respectively. Furthermore, the SCI lesion area volumes (Figure 1b) in the Sham, SCI, and SCI+ nesfatin‐1 groups were 0, 5.6, and 3.1 mm^3^, respectively. Moreover, the spinal cord water content (Figure 1c) on the 28th day postmodeling was greatly promoted from 59.3% to 83.9% in SCI mice, then reduced to 68.5% by nesfatin‐1. Collectively, the neurological state in SCI mice was improved by nesfatin‐1.

### 3.2 Nesfatin‐1 attenuated oxidative stress in SCI mice

**FIGURE 2:**
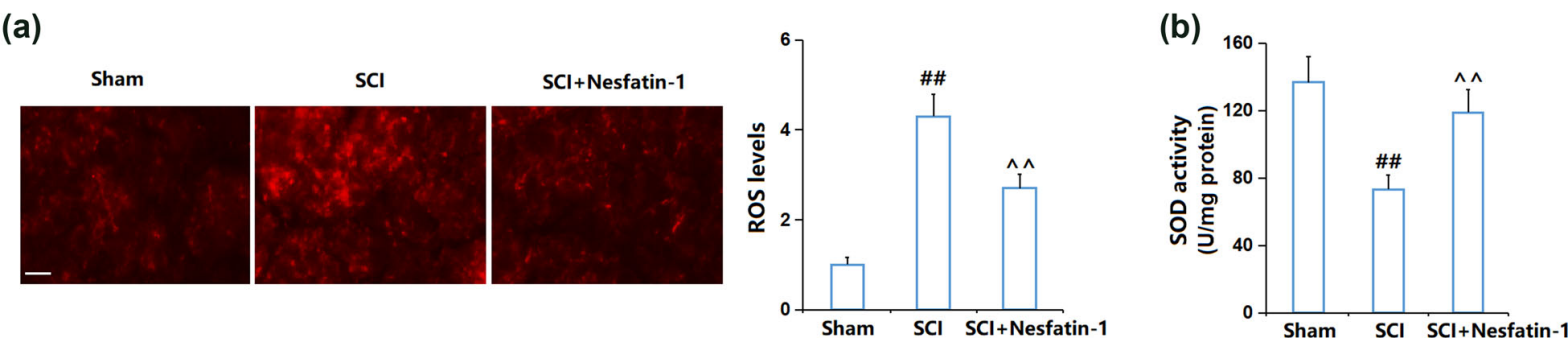
Nesfatin‐1 attenuated oxidative stress in SCI mice. (a) ROS levels were assayed by dihydroethidium (DHE) staining in SCI area; scale bar, 200 μm; (b) SOD activity in SCI area (^##^ *P* < .01 vs. vehicle group; ^^*P* < .01 vs. SCI group)

Oxidative stress is one of the main pathological characteristics of SCI (Z. Li et al., 2019). We found that the ROS level (Figure 2a) was extremely elevated in SCI mice, but greatly declined in the SCI + nesfatin‐1 group. Furthermore, the SOD activity (Figure 2b) declined from 136.7 U/mg protein to 73.2 U/mg protein, then reversed to 118.9 U/mg protein by nesfatin‐1. These data suggest that the oxidative stress in SCI mice was attenuated by nesfatin‐1.

### 3.3 Nesfatin‐1 prevented neuronal apoptosis in SCI mice on day 14

**FIGURE 3:**
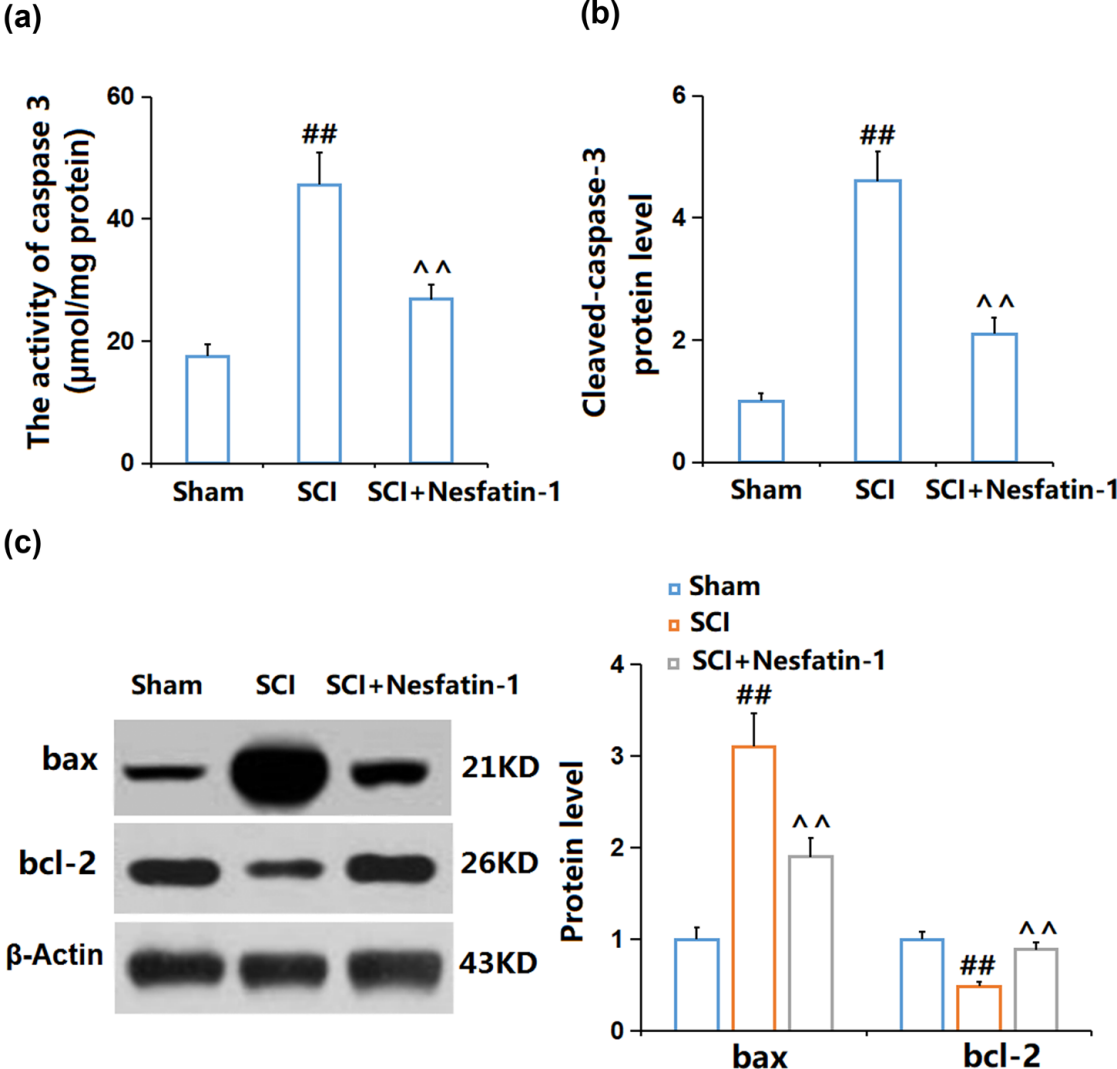
Nesfatin‐1 prevented neuronal apoptosis in mice who underwent SCI on day 14. (A) The activity of caspase 3; (b) immunofluorescence staining for cleaved‐caspase‐3; (C) Western blot for Bax and Bcl‐2 expression (^##^ *P* < .01 vs. vehicle group; ^^*P* < .01 vs. SCI group)

Subsequently, the protective effect of nesfatin‐1 on neurons was checked. Compared to the Sham group, the activity of caspase‐3 (Figure 3a) in SCI mice was promoted from 17.5 μmol/mg protein to 45.6 μmol/mg protein, which was greatly reduced to 26.8 μmol/mg protein by nesfatin‐1. In addition, cleaved‐caspase‐3 (Figure 3b) and Bax (Figure 3c) were found significantly upregulated, while Bcl‐2 was found downregulated in the SCI group, later dramatically rescued by nesfatin‐1, suggesting its protective property against neuronal apoptosis in SCI mice.

### 3.4 Nesfatin‐1 reduced the expression levels of proinflammatory cytokines in the SCI area

**FIGURE 4:**
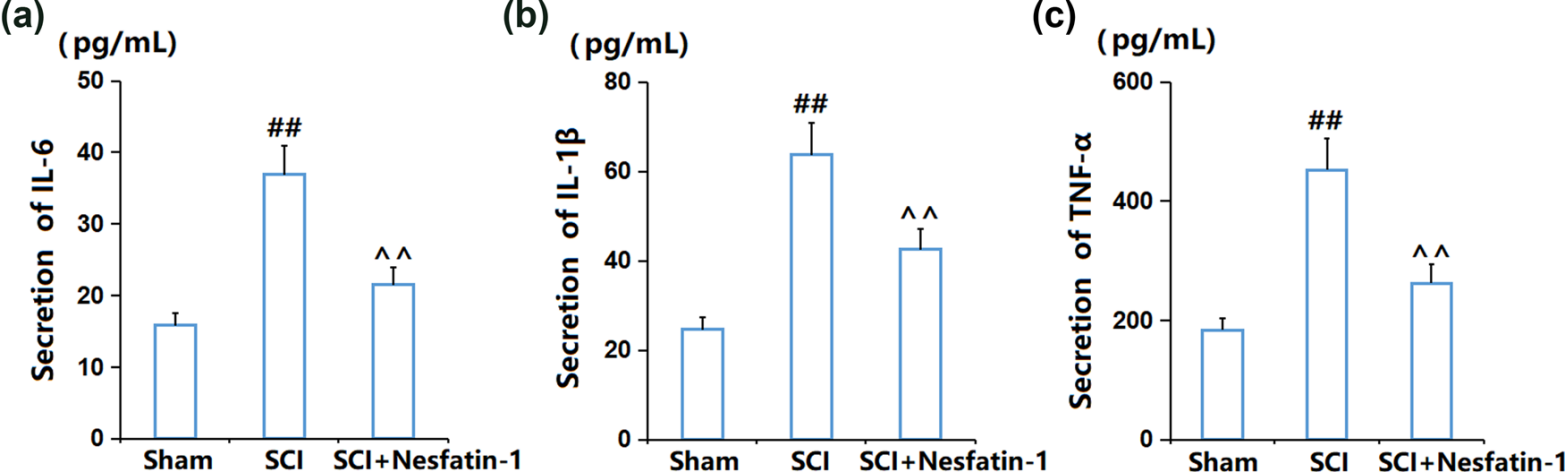
Nesfatin‐1 reduced the expression of pro‐inflammatory cytokines in the SCI area. (a) The levels of IL‐6; (b) The levels of IL‐1β; (c) The levels of TNF‐α (^##^ *P* < .01 vs. vehicle group; ^^*P* < .01 vs. SCI group)

The concentrations of inflammatory cytokines in SCI mice were subsequently detected. The release of IL‐6 was elevated from 15.8 pg/ml to 36.9 pg/ml in SCI mice (Figure 4a), then decreased to 21.5 pg/ml by nesfatin‐1. Furthermore, the concentrations of IL‐1β (Figure 4b) in the Sham, SCI, and SCI + nesfatin‐1 groups were 24.6, 63.7, and 42.5 pg/ml, respectively. Moreover, the level of TNF‐α (Figure 4c) was increased from 183.5 pg/ml to 452.3 pg/ml, which was then decreased to 262.1 pg/ml by nesfatin‐1. These results suggest an inhibitory effect of nesfatin‐1 in SCI mice.

### 3.5 Nesfatin‐1 promoted M2 microglia activation and inhibited M1 microglia activation in SCI mice

**FIGURE 5:**
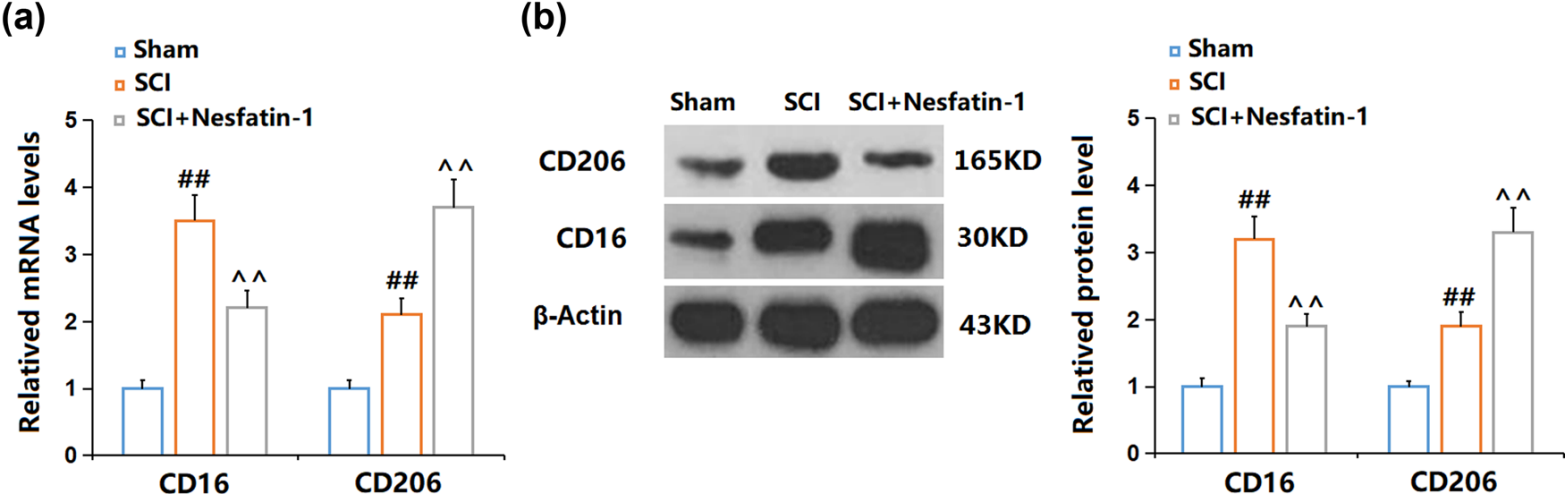
Nesfatin‐1 promoted M2 microglia activation and inhibited M1 microglia activation in SCI mice. (A). mRNA levels of M1 microglia marker CD16 and M2 Marker CD206; (B). Protein levels of M1 microglia marker CD16 and M2 Marker CD206 (^##^ *P* < .01 vs. vehicle group; ^^*P* < .01 vs. SCI group)

To further explore the regulatory mechanism of the anti‐inflammatory effect of nesfatin‐1, the polarization of microglia in SCI mice was checked. We found that CD16 (Figure 5), the biomarker of M1 microglia, was upregulated in SCI mice, then dramatically downregulated by nesfatin‐1. In addition, CD206, the biomarker of M2 microglia, was extremely upregulated in SCI mice, the expression of which was further elevated by nesfatin‐1. These data indicate that the M2 polarization of microglia was facilitated and the M1 polarization inhibited by nesfatin‐1 in SCI mice.

### 3.6 Nesfatin‐1 enhanced the phosphorylation of STAT6 in SCI mice

**FIGURE 6:**
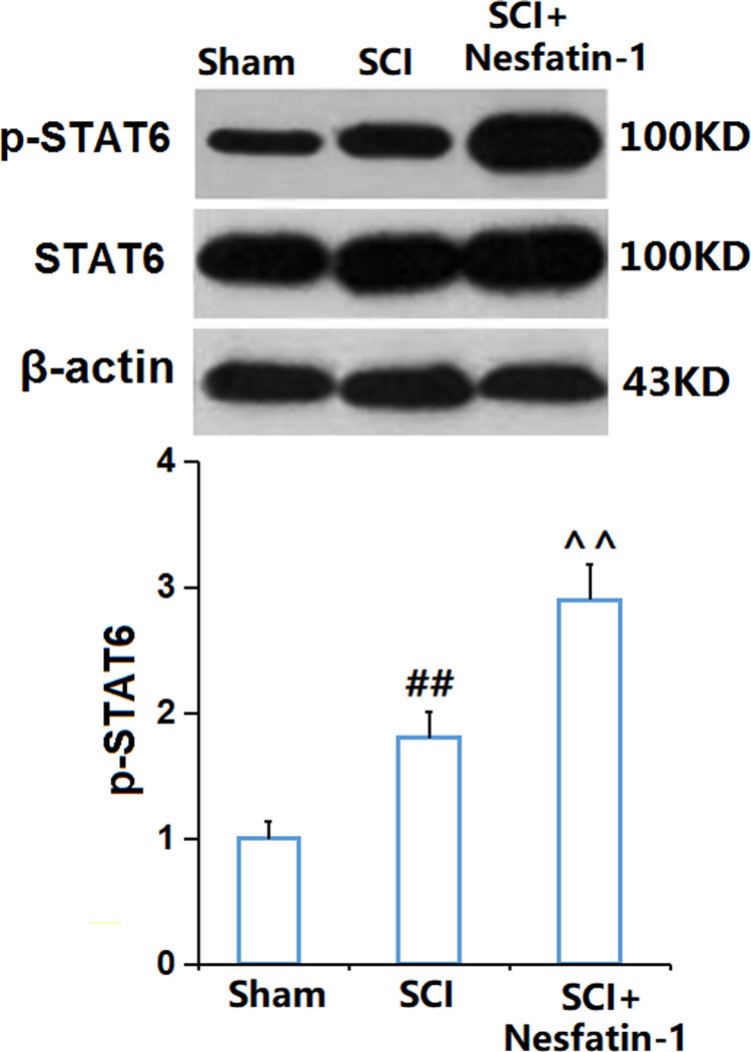
Nesfatin‐1 enhanced the phosphorylation of STAT1 in SCI mice. The levels of p‐STAT1 were measured by western blot analysis (^##^ *P* < .01 vs. vehicle group; ^^*P* < .01 vs. SCI group)

STAT6 is reported to drive the M2 polarization of microglia (Yang et al., 2017). We found that the expression level of p‐STAT6 was greatly elevated in SCI mice and was further promoted by nesfatin‐1 (Figure 6), suggesting that the activation of M2 microglia by nesfatin‐1 in SCI mice might be associated with the phosphorylation of STAT6.

### 3.7 Nesfatin‐1 prevented the activation of NF‐κB signaling in SCI mice

**FIGURE 7:**
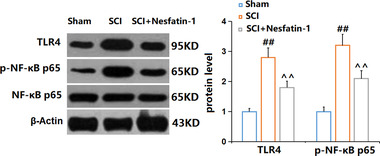
Nesfatin‐1 prevented activation of NF‐κB signaling in SCI mice. The levels of TLR4 and p‐NF‐κB p65 were measured by Western blot analysis (^##^ *P* < .01 vs. vehicle group; ^^*P* < .01 vs. SCI group)

The NF‐κB pathway is an important inflammatory signaling pathway involved in the pathogenesis of SCI (Liu et al., 2021). As illustrated in Figure 7, TLR‐4 and p‐NF‐κB p65 were found extremely upregulated in SCI mice, but dramatically rescued by Nesfatin‐1, suggesting an inhibitory property of Nesfatin‐1 on TLR4/NF‐κB signaling.

## 4 DISCUSSION

Within hours of SCI onset, the resident microglia at the injury site or those recruited by the surrounding spinal cord tissues are polarized into the M2 phenotype under pathological stimulation (Hu et al., 2015). The excessive immune/inflammatory response is repressed by M2 microglia via secreting anti‐inflammatory cytokines and neurotrophic factors and engulfing cell debris to protect the surrounding normal tissues. Approximately 1 week after SCI, rapid proliferation of microglia is observed (Bellver‐Landete et al., 2019). Under the continuous stimulation of the abnormal microenvironment in the injured spinal cord, M1 microglia gradually dominate to reach the polarization peak (Petralla et al., 2021). M1 microglia play the roles of inducing neurotoxicity, aggravating secondary spinal cord injury, and hindering nerve regeneration and reshaping, the process of which is sustainable for several weeks. The rapid transformation from M2 to M1 polarization following SCI is one of the important reasons for poor neurological recovery after SCI (Kobashi et al., 2020). The present study established that SCI mice models and their pathological states were identified by declined BBB scores, enlarged lesion areas, increased spinal cord water content, activated oxidative stress, aggravated neuronal apoptosis, and elevated production of proinflammatory cytokines, consistent with the pathological changes described previously (N. He et al., 2021; Z. Li et al., 2019; Sun et al., 2021). After the treatment with nesfatin‐1, these pathological changes were dramatically rescued, indicating a potential protecting property of nesfatin‐1 against SCI. Furthermore, both M1 and M2 polarization were observed in SCI mice, suggesting that the transformation from M2 to M1 polarization of microglia was in progress, which is in accordance with the report by Tao (Gaojian et al., 2020). Following the treatment with nesfatin‐1, the M1 polarization was repressed and the M2 polarization facilitated, suggesting that the transformation from M2 to M1 polarization of microglia in SCI mice was inhibited by nesfatin‐1.

STAT is an intracytoplasmic transcription factor, and currently, seven STAT proteins have been identified in mammals, including STAT1, STAT2, STAT3, STAT4, STAT5A, STAT5B, and STAT6. The seven members share five coiled‐coil and DNA‐binding domains, including the N‐terminal, coiled‐coil, DNA‐binding, Src homology 2, and C‐terminal transactivation domains (Villarino et al., 2020). The IL‐4 receptor, a type I cytokine receptor, contributes to the phosphorylation of JAK1 and JAK3, which leads to the phosphorylation of STAT6 (Banerjee et al., 2017). It is reported that the M2 polarization of microglia can be triggered by IL‐4 through the JAK1‐STAT6 pathway (Y. He et al., 2020). Y. He et al. (2020) showed that IL‐4 induced the transformation of microglia from the M1 to the M2 phenotype through the JAK1‐STAT6 pathway, and alleviated nerve damage in cerebral hemorrhage. We preliminarily found that the elevated expression level of p‐STAT6 in SCI mice was further increased by nesfatin‐1, consistent with the change of microglia to the M2 phenotype, suggesting that nesfatin‐1 might induce the polarization of M2 microglia by activating STAT6. Our future work will identify the underlying mechanism by coadministering nesfatin‐1 and an inhibitor of STAT6 into SCI mice. TLR‐4/NF‐κB signaling is an important inflammatory pathway observed in multiple diseases, including SCI (Wang et al., 2021). The activation of NF‐κB is triggered by the stimulation of TLR‐4, which then contributes to the phosphorylation of NF‐κB p65 and the transfer of p‐NF‐κB p65 from the cytoplasm to the nucleus. As a consequence, the transcription of multiple proinflammatory factors is activated to induce severe inflammation (Dejban et al., 2021). Recently, it is reported that the M1 polarization of microglia can be driven by NF‐κB signaling (R. Li et al., 2022). We found that the TLR‐4/NF‐κB pathway was significantly activated in SCI mice, in accordance with the results reported by Wang et al. (2021). After the treatment with nesfatin‐1, TLR‐4/NF‐κB signaling was greatly repressed, consistent with the changes in M1 polarization, suggesting that the M1 polarization of microglia was inhibited by nesfatin‐1 possibly via blocking TLR‐4/NF‐κB signaling. Our future work will identify the underlying mechanism by co‐administering nesfatin‐1 and an agonist of NF‐κB signaling into SCI mice.

Collectively, our data reveal that nesfatin‐1 exerted neuroprotective effects in SCI by promoting the activation of M2 microglia, and the underlying mechanisms might be related to the activation of STAT1 and inhibition of the TLR4/NF‐κB signaling pathway.

## AUTHOR CONTRIBUTIONS

Xin Jin and Huihui Dong designed the experiments; Xin Jin, Kai Guan, Zhengyu Chen, Yongwei Sun, Hongjun Huo, and Jinle Wang performed the experiments and collected the data; Huihui Dong analyzed the results and prepared the manuscript.

## CONFLICT OF INTEREST

None declared.

### PEER REVIEW

The peer review history for this article is available at: https://publons.com/publon/10.1002/brb3.2778.

## Data Availability

The data that support the findings of this study are available from the corresponding author upon reasonable request.
